# The developmental origins of hoarding disorder in adolescence: a longitudinal clinical interview study following an epidemiological survey

**DOI:** 10.1007/s00787-020-01527-2

**Published:** 2020-04-18

**Authors:** Volen Z. Ivanov, David Mataix-Cols, Eva Serlachius, Gustaf Brander, Anders Elmquist, Jesper Enander, Christian Rück

**Affiliations:** Department of Clinical Neuroscience, Centre for Psychiatry Research, Karolinska Institutet, Stockholm Health Care Services, Stockholm County Council, Karolinska University Hospital, SE 171 76 Huddinge, Sweden

**Keywords:** Hoarding disorder, Adolescence, Developmental, Twin study, Diagnostic criteria

## Abstract

Hoarding disorder (HD) is hypothesized to originate in childhood/adolescence but little is known about the presentation of hoarding symptoms in youth and their natural history. In this longitudinal study, we tracked and conducted in-depth psychiatric interviews with twins who participated in an epidemiological survey and screened positive on a measure of hoarding symptoms at age 15. Twins screening positive for clinically significant hoarding symptoms at age 15 (*n* = 42), their co-twins (*n* = 33), a group of screen negative twins (*n* = 49), and their parents underwent a clinical assessment a median of 3 years after the initial screening. The assessment included psychiatric screening, hoarding symptoms and cognitions, in-home or photographic assessment of clutter levels, parental accommodation and familial burden. None of the participants had significant levels of clutter at follow-up and thus did not meet strict criteria for HD. However, twins meeting partial criteria (i.e., DSM-5 criteria A and B) for HD (*n* = 28) had more psychiatric disorders and scored significantly higher on all measures of hoarding symptoms including researcher-rated levels of clutter in their homes, compared to twins who did not meet partial criteria for HD (*n* = 46). As currently defined in DSM-5, HD may be rare in young people. A non-negligible proportion of young people who were screen positive on hoarding symptoms at age 15 had substantial hoarding symptoms and other psychopathology at follow-up. Whether and how many of these individuals will develop full-blown HD is unknown but the results offer unique insights about the probable origins of HD in adolescence.

**Electronic supplementary material:**

The online version of this article (10.1007/s00787-020-01527-2) contains supplementary material, which is available to authorized users.

## Introduction

Hoarding disorder (HD) is characterized by a persistent difficulty discarding one’s possessions due to a perceived need to save the possessions and distress associated with discarding [1]. These difficulties result in severely cluttered living spaces, distress and/or functional impairment, which impact their quality of life and that of family members and the wider community [2, 3]. Prevalence estimates indicate that HD affects approximately 2.5% of the working-age population [4, 5] with some evidence suggesting that it becomes more prevalent with increasing age [6].

Although most individuals who seek help or come to the attention of services are middle-aged or elderly, the origins of hoarding difficulties are likely to be found in childhood. Retrospective reports of adult individuals with HD consistently suggest that the first symptoms appeared in childhood and remained mild during adolescence and young adulthood, before deteriorating and remaining chronic throughout the lifespan [7–9]. These retrospective reports also suggest that the most impairing feature of HD, the accumulation of clutter, begins to cause clinically significant impairment by the third decade of life [7–10]. However, investigations of hoarding symptoms in childhood and adolescence have been rare [11, 12], and longitudinal investigations across the lifespan are lacking.

Recent community-based work has shown that hoarding symptoms indeed occur during childhood and adolescence, and may be fairly prevalent (estimates widely ranging from 2 to 9% of the population) [6, 10, 13]. A number of studies have also examined the correlates of hoarding symptoms in treatment-seeking samples of youth with obsessive–compulsive disorder (OCD) [14, 15], attention-deficit/hyperactivity disorder (ADHD) [16, 17], autism spectrum disorders (ASD) [18] and anxiety disorders [19], reporting high rates of hoarding symptoms in all these clinical groups. However, ‘caseness’ definitions have varied and since these estimates are based on self-report, it is unclear how many of these individuals would meet diagnostic criteria for HD. Further insights into the clinical presentation of hoarding symptoms in youth come from a limited number of case reports, which suggest that the core features of HD (difficulties discarding possessions due to a perceived need to save them and associated distress with discarding them are indeed present in childhood and adolescence. However, other features such as excessive acquisition and clutter are thought to be much less common and prominent in children [10, 20, 21]. Parental control over living spaces and restricted freedom while living at home, are factors that likely alter and limit, the features and consequences of hoarding behavior in young people [22].

In general, previous research on childhood hoarding has suffered from a number of methodological limitations, including the study of hoarding features in the context of other disorders, such as OCD or ADHD, and the fact that no studies employed current DSM-5 criteria to diagnose cases. It is, therefore, unclear how informative these previous studies are and whether the DSM-5 criteria are helpful in non-adult populations. An improved understanding of the developmental origins of hoarding symptoms in childhood and adolescence has the potential to influence the refinement of the diagnostic criteria and more importantly, given the chronicity and deleterious long-term effects of HD, to inform future prevention and intervention strategies.

This prospective longitudinal study aimed to further understand the features, family impact and natural history of hoarding symptoms in youth. We followed-up and interviewed a group of twins who participated in a large, population-based study [10] and screened positive for clinically significant hoarding symptoms at age 15. Specifically, these individuals, their co-twins, screen negative twins and their parents underwent a detailed psychiatric evaluation 1–6 years (median 3 years) after their initial screening, including the assessment of hoarding symptoms and cognitions, clutter levels, parental accommodation of symptoms and familial burden.

## Methods

### Participants

Study participants were 15-year old monozygotic (MZ) and dizygotic (DZ) twins and their parents from the Swedish Twin Registry taking part in the longitudinal Child and Adolescent Twin Study in Sweden (CATSS). Starting in 2004, the parents of all twins in Sweden born 1992 and onward were contacted when they turned 9 or 12 years [23]. The first follow-up wave of CATSS included 8455 twins from all over Sweden born 1994–1996 and who had turned 15 (CATSS-15). All these twins were screened for hoarding symptoms using the Hoarding Rating Scale-Self Report (HRS-SR) [24], a widely used measure of hoarding symptoms that possesses excellent psychometric properties. A total of 3974 twins returned the questionnaires. Participants who scored at least moderately (a score of 4 or higher out of 8) on items measuring clutter, difficulties discarding and distress and/or impairment were considered to have clinically significant hoarding symptoms based on established cut-off values [24, 25]. Additional information about the sample can be found in the original publication [10]. In the present study, all twins who screened positive for significant hoarding symptoms at age 15 (*n* = 79; 2% of the screened sample), along with screen negative co-twins (*n* = 67) and one parent to every twin pair were contacted and offered participation in a face-to-face assessment, which took place 1–6 years (median = 3 years) after the initial screening. For comparison, we also randomly selected and contacted 76 screen-negative twins from CATSS-15. Screen-negative individuals were defined as scoring < 4 on HRS-SR items measuring difficulties discarding, clutter in one’s room, distress and impairment.

A total of 42 (53%) of all screen positive twins, 33 of their co-twins (49% of all co-twins) and 49 screen-negative (64% of all screen negative) and their parents agreed to participate in the follow-up interviews. Overall, there were few differences between participants and non-participants. No differences emerged in terms of zygosity and rates of ADHD, ASD and OCD (*p* values ranging from 0.30 to 1.00). However, participants from the screen-positive group who took part in the current study had significantly higher HRS-SR total scores compared to those who declined participation (19.1 vs. 14.1; *t* = − 3.31, *df* = 107, *p* < 0.05). Furthermore, in the screen negative group, there were significantly more boys among individuals who declined participation (48% vs. 25%; *χ*^2^ = 4.751, *df* = 1, *p* < 0.05). Participant flow throughout the stages of screening and assessment are displayed in Fig. 1 (Supplementary material).

### Procedures

All eligible participants and their parents were first contacted by post with information regarding the present study. Shortly thereafter, the twins were contacted via telephone and offered participation in the study. Assessments of the screen positive twins were conducted at either a test center in Stockholm, Sweden, a test center within the vicinity of their homes or in their homes, depending on the twin’s preference. Participants hailed from several locations across Sweden (see Fig. 2, Supplementary material) and the research team travelled to these locations to conduct the interviews. Participants who chose to not be assessed in their home were asked to provide representative photos of their bedroom. Twins who screened negative at age 15 were assessed over the telephone and were mailed self-report questionnaires. Parent assessments were conducted face-to-face or over the telephone. All assessments were carried out by two clinical psychologists and two graduate students majoring in psychology. All participants received gift vouchers of 500 SEK (50 USD) for their participation and took part in a competition to win an iPad. Ethical approval was obtained from the Regional Ethics Review Board in Stockholm (reference numbers: 2012/471-31/2, 2012/1673-32 and 2014/160-32). Written informed consent was obtained from all twins and their parents prior to their participation.

### Measures

#### Researcher administered

Participants were screened for psychiatric diagnoses with the Schedule for Affective Disorders and Schizophrenia for School-Age Children Present and Lifetime Version (K-SADS-PL) [26]. The Structured Interview for Hoarding Disorder (SIHD) was administered to diagnose HD. This instrument probes the six core criteria of HD as well as its two specifiers (excessive acquisition and level of insight) [27, 28]. Assessors based their judgement of whether a participant endorsed the diagnostic criteria on examination of participants’ reports and direct observation of the participant’s dwellings (when home visits were possible), and did not have access to current self-report measures at the time of assessment. The assessors rated the amount of clutter in the participant’s bedrooms using the Clutter Image Rating (CIR) [29]. Assessors also administered a shortened version of a hoarding interview developed by Pertusa et al. [30]. The instrument contains questions about types of saved items from a list of everyday objects and reasons for saving.

#### Self-reported measures

Hoarding symptoms were measured using the Saving Inventory-Revised (SI-R) [31] and the Hoarding Rating Scale-Self-Report (HRS-SR) [24]. In the present study, a modification was made to the clutter item of the HRS-SR to reflect the living situation of the responder referring to the persons own room instead of the entire home, unless the adolescent or young adult lived on their own. To examine hoarding beliefs, we used the Saving Cognitions Inventory (SCI) [32]. Autistic traits were self-rated with the Adult Autism Spectrum Quotient (AQ) [33] and the Brown Attention-Deficit Disorder Scales (BADDS) were used to measure executive function impairments typical for ADHD [34].

#### Parent-rated measures

The Children’s Saving Inventory (CSI) is a 23-item parent-rated instrument assessing child hoarding behaviors including acquisition, difficulty discarding, clutter, distress and impairment [35]. Levels of parental accommodation to twin hoarding behaviors (e.g., avoiding to discard the twins’ possessions) and family burden were measured with the Family Impact Scale for Hoarding (FISH) [36]. Parents to all twins were also interviewed with the parent versions of the ADHD and ASD sections of K-SADS-PL.

### Statistical analyses

Data were analyzed with STATA 13 (STATA Corporation, College Station, TX). As some of the individuals in our analyses were related (twin pairs), we used linear regression with a robust sandwich estimator option in STATA [vce (cluster)] to examine between-group differences on continuous variables (e.g., symptom levels of hoarding, ADHD and ASD) in the group comparisons. This option relaxes the requirement of independent observations when estimating standard errors and requires that observations are only independent across twin pairs (clusters) but not within twin pairs. Categorical data (e.g., gender, educational level and presence of comorbid diagnosis) were compared using chi-square tests and Fisher’s exact test. All statistical tests were two-tailed and the significance level was set to *p* < 0.05.

## Results

### Endorsement of HD criteria

The proportion of participants from the screen positive and screen negative groups endorsing each of the diagnostic criteria and the two specifiers for HD are displayed in Table 1. As expected, participants from the screen-positive group were more likely to endorse almost all criteria for HD indicating an association between screening status at age 15 and endorsement of diagnostic criteria at the clinical interview. However, none of the participants were judged to have significant clutter in their rooms (DSM-5 criterion C) and, for that reason, none of the participants met full criteria for HD.

**Table 1:** Endorsement of diagnostic criteria and specifiers for HD based on initial screening status

DSM-5 criterion	Group			*p* value
	Screen positive (*N* = 42)	Screen positive co-twins (*N* = 33)	Screen negative (*N* = 49)	
	*N* (%)	*N* (%)	*N* (%)	
A	20 (47.6)	8 (24.2)	4 (8.2)	< 0.000
B	17 (40.5)	8 (24.2)	3 (6.1)	< 0.000
C	0 (0.0)	0 (0.0)	0 (0.0)	N.A
D	9 (21.4)	2 (6.1)	1 (2.0)	< 0.05
E^a^	17 (40.5)	8 (24.2)	3 (6.1)	< 0.000
F^a^	17 (40.5)	8 (24.2)	3 (6.1)	< 0.000
Specifier				
Excessive acquisition	6 (14.3)	1 (3.0)	3 (6.1)	0.1681
Insight	1 (2.4)	0 (0.0)	0 (0.0)	0.4355

Criterion A: Persistent difficulty discarding or parting with possessions, regardless of their actual value

Criterion B: This difficulty is due to a perceived need to save items and to distress associated with discarding them

Criterion C: The difficulty discarding possessions results in the accumulation of possessions that congest and clutter active living areas and substantially compromises their intended use

Criterion D*:* The hoarding causes clinically significant distress or impairment in social, occupational, or other important areas of functioning (including maintaining a safe environment for self and others)

^a^Only relevant for those endorsing criteria A and B

Of the 42 participants in the screen-positive group, almost half (*n* = 20; 47.6%) endorsed DSM-5 criterion A (difficulties discarding or parting with possessions), while only 8 (24.2%) of the co-twins and 4 (8.2%) from the screen negative group endorsed this criterion (Table 1). Most participants (17/20) from the screen-positive group who met criterion A also met criterion B (difficulties discarding due to a perceived need to save the item or distress associated with discarding). However, three individuals from the screen-positive group reported difficulties letting go of possessions due to low motivation to clean out old items from their room, rather than needing to save these items or distress when discarding, and thus did not meet criterion B. Despite the general mild levels of clutter, 12 participants (nine from the screen-positive group, two co-twins and one from the screen-negative group) reported experiencing distress and/or impairment as a direct result of their hoarding behaviors. Reasons mentioned were being ashamed of these behaviors or due to the disorganization of things in their room caused by their hoarding. Finally, all participants who met criteria A and B also endorsed criteria E and F since their difficulties were deemed not to be the result of any alternative somatic or psychiatric conditions.

With regard to the HD specifiers, participants in the screen-positive group were twice as likely to endorse the excessive acquisition specifier compared to the screen negative group and almost five times as likely compared to the co-twins (Table 1). The between-group differences were however not statistically significant due to small numbers. Only one person in the screen-positive group had a poor insight.

### Hoarding vs comparison groups

Based on criteria endorsement at the diagnostic interview, we categorized the participants into two new groups (hereafter the *hoarding group* and the *comparison group*) for all further analyses. The hoarding group (*n* = 28) consisted of participants from the original screen-positive group (*n* = 17), their screen negative co-twins (*n* = 8) and screen negative controls (*n* = 3) who reported persistent difficulties parting with possessions (criterion A) due to a perceived need to save the items and distress associated with discarding (criterion B) at the time of the interview. Participants from the screen-negative group who endorsed none of the diagnostic criteria for HD or only met criterion A were included in the comparison group (*n* = 46).

### Demographic variables

Table 2 displays the demographic and clinical characteristics for participants in the hoarding and the comparison groups. Participants in both groups were evenly matched and predominantly included girls and students. However, the mean age in the comparison group was slightly higher than in the hoarding group (18.2 vs. 19.0 years, *t* = 3.08, *df* = 72, *p* < 0.05). This difference likely occurred because participants in the screen negative group were contacted for clinical assessments some time after the screen-positive group (for matching reasons). However, age did not correlate with hoarding symptom severity at the time of the clinical assessments as measured with the SI-R and the HRS-SR in either group (*p* values ranging from 0.06 to 0.54). For this reason, age was not considered further in any subsequent analyses.

**Table 2:** Demographic characteristics and psychiatric disorders in twins endorsing criteria A and B for hoarding disorder (hoarding group) compared to twins who did not endorse both criteria (comparison group)

Variable	Group		Test stat	*p* value
	Hoarding (*N* = 28)	Comparison (*N* = 46)		
	*N* (%)	*N* (%)		
Gender, *n* (%)				
Female	23 (82.1)	35 (76.1)	0.3767	0.539
Age, mean (SD)	18.2 (1.2)	19.0 (0.9)	3.0787	< 0.05
Occupation, *n* (%)				
Student	22 (78.6)	28 (60.9)	2.4889	0.115
Working	4 (14.3)	13 (28.3)		0.2548
Unemployed	2 (7.1)	4 (8.7)		1.000
Diagnosis, *n* (%)				
Mood disorder (current)	3 (10.7)	0 (0.0)		0.051
Mood disorder (lifetime)	14 (50.0)	2 (4.3)		< 0.000
Anxiety disorder (current)	15 (53.6)	4 (8.3)		< 0.000
Anxiety disorder (lifetime)	5 (17.9)	0 (0.0)		< 0.05
Eating disorder (current or lifetime)	2 (7.1)	4 (8.7)		1.00
OCD (current or lifetime)	1 (3.6)	0 (0.0)		0.378
ADHD (current or lifetime)	1 (3.6)	0 (0.0)		0.378
ODD (current or lifetime)	2 (7.1)	0 (0.0)		0.14
Any current or lifetime	22 (78.6)	7 (17.4)	29.3147	< 0.000

*OCD* obsessive–compulsive disorder, *ADHD* attention-deficit/hyperactivity-disorder, *ODD* oppositional defiant disorder

### Psychiatric diagnoses

As shown in Table 2, most psychiatric disorders were more likely to occur in the hoarding group. Twenty-two (78.6%) of the 28 participants in the hoarding group had at least one current or lifetime psychiatric disorder compared to seven (17.4%) in the comparison group. The most frequent current diagnosis in the hoarding group was an anxiety disorder (*n* = 15; 53.6%) while 14 participants (50.0%) reported a past episode of major depression. Notably, only one participant in the hoarding group met criteria for OCD (lifetime). Finally, self-reported symptoms of ADHD and ASD were both significantly higher in the hoarding group (Table 3).

**Table 3:** Comparison of hoarding symptom severity, hoarding cognitions, family accommodation and family burden in the hoarding and comparison groups

Measure	Rater	Group		*F*	*df*	*p* value
		Hoarding	Comparison			
		(*N* = 28)	(*N* = 46)			
		Mean (SE)	Mean (SE)			
SIR total	Twin	30.79 (2.02)	9.83 (2.66)	61.97	49	< 0.000
SIR clutter	Twin	8.68 (0.97)	3.39 (1.24)	18.04	49	< 0.000
SIR discarding	Twin	14.00 (0.79)	3.43 (1.06)	99.25	49	< 0.000
SIR acquisition	Twin	7.89 (0.81)	3.00 (0.92)	28.14	49	< 0.000
CSI total	Parent	24.60 (3.79)	12.04 (4.75)	6.98	28	< 0.05
CSI discarding	Parent	9.35 (1.42)	2.92 (1.65)	15.13	28	< 0.000
CSI clutter	Parent	6.55 (1.01)	3.88 (1.43)	3.49	28	0.072
CSI acquisition	Parent	4.25 (1.16)	3.28 (1.46)	0.44	28	0.5110
CSI dist/imp	Parent	4.35 (0.62)	1.96 (0.75)	10.08	28	< 0.05
CIR	Researcher	1.92 (0.11)	1.13 (0.13)	37.37	41	< 0.000
HRS-SR	Twin	12.64 (1.20)	2.89 (1.47)	43.98	49	< 0.000
SCI	Twin	70.29 (6.17)	40.50 (7.23)	16.96	49	< 0.000
FISH A	Parent	3.50 (0.91)	0.63 (0.98)	8.58	29	< 0.05
FISH B	Parent	0.05 (0.48)	0.43 (0.45)	0.74	28	0.3979
BADDS	Twin	53.19 (3.59)	26.56 (4.68)	32.33	46	< 0.000
AQ	Twin	17.56 (1.86)	11.46 (2.05)	8.87	48	< 0.05

*SI-R* saving inventory-revised, *CSI* children’s saving inventory, *CIR* clutter image rating, *HRS-SR* hoarding rating scale-self report, *SCI* saving cognitions inventory, *FISH A* family impact scale for hoarding-accommodation subscale, *FISH B* family impact scale for hoarding-burden subscale, *BADDS* Brown’s ADD scales, *AQ* adult autism spectrum quotient

### Hoarding symptom severity

As shown in Table 3, participants in the hoarding group had significantly higher scores than the comparison group on all self- and parent-rated hoarding symptom measures except the clutter and excessive acquisition subscales of the CSI. Inspection of SI-R subscales revealed that scores on the difficulty discarding subscales were highest while excessive acquisition and clutter were less prominent, but still significant. The higher levels of self-reported clutter in the hoarding group relative to the comparison group were corroborated by investigator ratings of clutter in the participants’ rooms using the CIR.

### Family impact

Parent-rated measures were available for 20 (71%) of the participants included in the hoarding group and for 25 (54%) of those included in the comparison group. Parents of twins in the hoarding group reported higher levels of accommodation but not burden associated with the young persons’ hoarding behaviors (Table 3).

### Saved items and reasons for saving

Table 4 shows the types of saved items and their frequency in the hoarding and comparison groups. In general, participants from both groups reported saving similar items with books, tickets and letters/postcards being most frequent. However, the proportion of participants who saved these items was generally higher in the hoarding group than in the comparison group. Furthermore, participants in the hoarding group reported saving a significantly greater number of different classes of items (8.8 vs 2.7, respectively).

**Table 4:** Types of saved items and number of different classes of saved items in the hoarding and comparison groups

Saved item	Group		*χ*^2^/Fisher’s	*p* value
	Hoarding (*N* = 28)	Comparison (*N* = 46)		
	*N* (%)	*N* (%)		
Books	19 (67.9)	11 (23.9)	13.94	< 0.000
Tickets	17 (60.7)	11 (23.9)	10.02	< 0.05
Letters/postcards	17 (60.7)	12 (26.1)	8.76	< 0.05
Old clothes	17 (60.7)	9 (19.6)	12.93	< 0.000
CD/DVDs	15 (53.6)	9 (19.6)	9.19	< 0.05
Pens	15 (53.6)	6 (13.0)	14.07	< 0.000
Old notes	14 (50.0)	2 (4.3)		< 0.000
Receipts	13 (46.4)	10 (21.7)	4.95	< 0.05
Magazines	13 (46.4)	4 (8.7)		< 0.000
Cardboard boxes	11 (39.3)	2 (4.3)		< 0.000
Stuffed animals	11 (39.3)	2 (4.3)		< 0.000
Old makeup	10 (35.7)	5 (10.9)		< 0.05
Bills	9 (32.1)	6 (13.0)	3.93	< 0.05
Video games	8 (28.6)	5 (10.9)		0.065
Plastic bags	7 (25.0)	5 (10.9)		0.192
Wool/fabric	5 (17.9)	0 (0.0)		< 0.05
Newspapers	3 (10.7)	1 (2.2)		0.149
Tools	3 (10.7)	0 (0.0)		0.051
Plastic containers	2 (7.1)	0 (0.0)		0.140
Old toys	2 (7.1)	0 (0.0)		0.140
School work	2 (7.1)	1 (2.2)		0.558
Electronic parts	2 (7.1)	0 (0.0)		0.140
Bodily products	1 (3.6)	0 (0.0)		0.378
Old food	1 (3.6)	0 (0.0)		0.378
Animals	0 (0.0)	0 (0.0)	NA	NA

	Mean (SD)	Mean (SD)	Student’s *t*	*p*
No. of different classes of saved items	8.8 (4.3)	2.7 (2.7)	7.3483	< 0.000

As shown in Table 5, participants from both study groups also largely reported similar reasons for saving. However, the most common reason for saving items in the hoarding group was due to a belief that the items could be useful in the future (*n* = 16; 57.1%) while saving due to a perceived sentimental value of the saved items was most commonly reported in the comparison group (*n* = 23; 50.0%). Participants in the hoarding group also reported significantly more different reasons for saving items than the comparison group (3 vs. 1, *t* = 4.38, *df* = 72, *p* < 0.000).

**Table 5:** Main reason for saving and number of different reasons for saving possessions in the hoarding and comparison groups

Main reason for saving	Group		*χ*^2^/Fisher’s	*p* value
	Hoarding (*N* = 28)	Comparison (*N* = 46)		
	*N* (%)	*N* (%)		
Useful in future	16 (57.1)	13 (28.3)	6.09	< 0.05
Sentimental value	10 (35.7)	23 (50.0)	1.44	0.231
Learned not to waste as a child	1 (3.6)	0 (0.0)		0.378
Things perceived as more than just objects	1 (3.6)	0 (0.0)		0.378
Part of identity	0 (0.0)	2 (4.3)		0.523
Monetary value	0 (0.0)	2 (4.3)		0.523
No reason for saving	0 (0.0)	6 (13.0)		0.077
Number of different reasons for saving (M, SD)	2.61 (1.81)	1.20 (0.96)	4.38	< 0.000

## Discussion

Hoarding behavior in youth is an under-researched phenomenon. Basic questions about the clinical phenomenology and natural history of hoarding symptoms in young people are largely unanswered. In this prospective study, we tracked a sample of twins screening positive for self-reported hoarding symptoms at age 15 and conducted in-depth interviews with them and their parents 3 years later. Our results showed that difficulties discarding due to a perceived need to save possessions and distress associated with discarding them (corresponding to diagnostic criteria A and B for HD in DSM-5) were present in a substantial proportion (40%) of participants who screened positive at age 15. However, none of the participants in our study presented with impeding levels of clutter (corresponding to diagnostic criterion C) at follow-up, even when taking parental involvement into account, and thus did not meet full criteria for HD.

Although the absence of impending levels of clutter in our sample was the reason why HD could not be diagnosed, even when the criteria for clutter and distress/impairment were waived, only 40% (17/42) of those screening positive for clinically significant hoarding symptoms at age 15 were considered to actually meet criteria A and B at the follow-up clinical assessments. Thus, the HRS-SR (the screening instrument used at age 15) had relatively poor predictive value. It is important to note that the HRS-SR was developed for its use in adult populations and that hoarding behavior in young people might be a slightly different phenomenon than hoarding in adults. It is possible that the HRS-SR, which was designed to screen and quantify the severity of hoarding symptoms, may partly capture developmentally normal behavior in youth. Thus, some of the screen-positive cases may have been false positives. Additionally, hoarding symptoms might be less stable in youth than they are in adults; case studies have suggested that hoarding symptoms in childhood/adolescence may be episodic [21], and a recent epidemiological follow-up of the twin cohort in the current study revealed that hoarding symptoms were only moderately stable (*r* = 0.40) between ages 15 and 18 [37]. Regardless, based on our results, it is plausible that impeding levels of clutter simply are rare during adolescence, even among young people with stable hoarding symptoms. Our results suggest that HD, as currently defined in DSM-5, might be far less common in young people than previously suggested by previous studies relying on self-report measures [6, 10, 13].

Although no participants meet full criteria for HD in our sample, by comparing those who met partial criteria for HD to those who did not, we were able to gain new insights into the clinical presentation and correlates of a group of non-treatment seeking young people with prominent hoarding symptoms. First, the assessment of diagnostic criteria endorsement was confirmed by all self- and parent-rated hoarding symptoms, which were higher for those who met partial criteria for HD (hoarding group). Based on the self-rated measures, and line with previous studies [10, 20, 21], difficulty discarding possessions was the most prominent feature in this group. Moreover, by including ratings of photographs of the participants’ rooms or carrying out assessments in their homes, we obtained more valid estimates of clutter [38] than in previous studies of young people. Our results showed that in youth with hoarding behaviors, the difficulties discarding possessions might already be resulting in heightened levels of clutter compared to non-hoarding youth. As expected, CIR scores in the hoarding group were lower than what is typically observed among community samples of adults with HD [4, 28] and support the data from retrospective adult reports suggesting that, for the average person with HD, levels of clutter first become impairing later in life [7]. Interestingly, and in contrast to previous studies suggesting that parents actively limit clutter in younger children [14, 21] participants in the hoarding group did not report that the lack of impeding clutter was due to parental involvement. This finding was also supported by parent-reported levels of accommodation, which albeit significantly higher than in the comparison group, were minimal compared to those reported by relatives and caretakers of adults with HD [2]. The notion that parental control over the adolescents’ space decreases as they grow older and become more independent, is however expected and resonates well with recent epidemiological findings indicating that shared environmental factors within twin pairs (e.g., parenting style) has an effect on hoarding symptoms at age 15 but not at ages 18 or 20–28 [37]. Our results suggest that late adolescence constitutes a period in development when possessions are saved and acquired without environmental influences posed by relatives or a spouse but have not yet accumulated to a degree that impairs function.

Analysis of the occurrence of psychiatric diagnoses revealed that participants in the hoarding group were more psychiatrically burdened than the comparison group. Moreover, the pattern of psychopathology was similar to that observed in adult samples, in which mood and anxiety disorders often co-occur with HD [4, 39, 40]. Notably, the finding that none of the participants in the hoarding group had co-occurring OCD, supports previous epidemiological reports [10, 13] indicating that hoarding symptoms present independently of OCD in a substantial proportion of adolescents. Furthermore, although, a diagnosis of ADHD was rare in any of the groups, symptoms of ADHD were common in the hoarding group. This is not surprising given the similarities in aspects of executive functioning (e.g., difficulties organizing and making decisions) common to both ADHD [41] and adult HD [42]. This finding also aligns with an emerging body of evidence suggesting a link between hoarding and ADHD in both children [16, 17, 43, 44] and adults [39, 43, 44]. A potential etiological overlap between hoarding and ADHD warrants further investigation and could potentially open up novel treatment avenues for HD. A potential link between hoarding and neuropsychiatric symptoms was also supported by the self-reported elevated symptoms of ASD in the hoarding group. This association might however not be specific to hoarding, but rather represent a link between ASD and general psychiatric burden, as has been suggested in adults with HD [45].

Examination of saving behaviors revealed that participants in the hoarding group saved a wide range of everyday items, reported more reasons for saving than the comparison group and that a possible future use of their objects was the most frequently stated reason for saving. This finding is in contrast to previous literature [21, 22] stating that young people with hoarding symptoms collect worthless objects. Instead, these results resemble findings in adults with HD, who commonly save both valuable and less valuable objects such as clothes, newspapers, books, receipts and sentimental objects [30, 46, 47]. The similarity to adults with HD was also supported by reports showing that individuals with HD report more reasons for saving compared to non-hoarders [48] and most commonly report saving due to seeing a possible future use for the items and due to sentimental reasons [28].

In adults, HD is widely acknowledged to be a chronic, debilitating disorder that is difficult to treat [49]. The results of our study question the validity of the current DSM-5 criteria in young people. A more developmentally nuanced definition of problematic hoarding than the current DSM-5 criteria for HD is needed to identify individuals who may later develop full-blown HD. One way of refining the criteria for HD in young people would be to relax the clutter criterion, as potentially at-risk individuals will not display obvious signs of obstructive clutter during adolescence. More research into the ‘early warning signs’ in adolescence is needed to inform potential prevention strategies. Whether parent-reported instruments, such as the CSI [35] have better predictive value than self-reported instruments, such as the one used here, is an interesting question for the future.

In terms of treatment implications, our results suggest that interventions for young people with hoarding difficulties primarily should address difficulties discarding objects, hoarding related cognitions and practice of organizational skills of belongings while management of large amounts of clutter, excessive acquisition and parental accommodation should not be prioritized in treatment.

Our study is the first prospective follow-up of youth who self-identified as having problematic hoarding behavior in adolescence. Strengths include the sample of non-selected and non-treatment seeking youth from the community and the use of multiple informants and in-depth interviews often conducted in the young person’s own home. Several limitations are also worth noting. First, the relatively short follow-up of approximately 3 years was clearly insufficient to determine if and how many of the participants from our sample would develop HD in the future. As HD is thought to gradually develop over several decades [7–9], such study would be difficult to conduct. Nevertheless, the striking similarities in phenomenology and comorbidity suggest that we captured a phenotype that may be a precursor to later-life, full-blown HD, at least in some individuals. Second, the difference in score on the HRS-SR between responders and non-responders in the screen negative group might have introduced bias; it could be argued, that twins with more severe hoarding symptoms would decline participation in the clinical assessments due to shame or fear of social stigmatization. However, since HRS-SR scores at age 15 were higher among those who participated in this study, it is more likely that we captured those with more severe current hoarding symptoms.

## Electronic supplementary material

Below is the link to the electronic supplementary material.

Supplementary file1 (DOCX 186 kb)
