# Supplementary material for: The developmental origins of hoarding disorder in adolescence: a longitudinal clinical interview study following an epidemiological survey
Source: Eur Child Adolesc Psychiatry. 2020 Apr 18;30(3):415–25. doi: 10.1007/s00787-020-01527-2 (PMC8019421; doi:10.1007/s00787-020-01527-2)
Supplement: Supplementary file 1 — Supplementary file1 (DOCX 186 kb) [file 787_2020_1527_MOESM1_ESM.docx]

**FIGURE 1** | Flow chart of study participants.

Twins contacted in present study (n=222)

**Declined participation in assessments (n=98)**

Screen positive (n=37) Screen negative co-twins (n=34)

Screen negative controls (n=27)

Hoarding group^a^

(n=28)

Comparison group^b^

(n=46)

Screen positive

(n=79)

Screen negative controls

(n=76)

Screen negative co-twins

(n=67)

Screen positive

(n=42)

Screen negative co-twins

(n=33)

Screen negative controls

(n=49)

Twins included in the group comparison analyses (n=74)

Twins assessed in present study (n=124)

**^a^** The hoarding group consisted of twins who met diagnostic criteria A and B for HD and comprised 17 twins who were screen positive at age 15, 8 of their co-twins who were screen negative and 3 twins (who thus crossed over) from the screen negative control group.

**^b^** The comparison group consisted of twins who did not meet criteria A and B for HD and comprised 46 twins who were all screen negative at age 15.

**FIGURE 2** | Map of Sweden depicting approximate locations of participants interviewed in the screen-positive hoarding group.

**
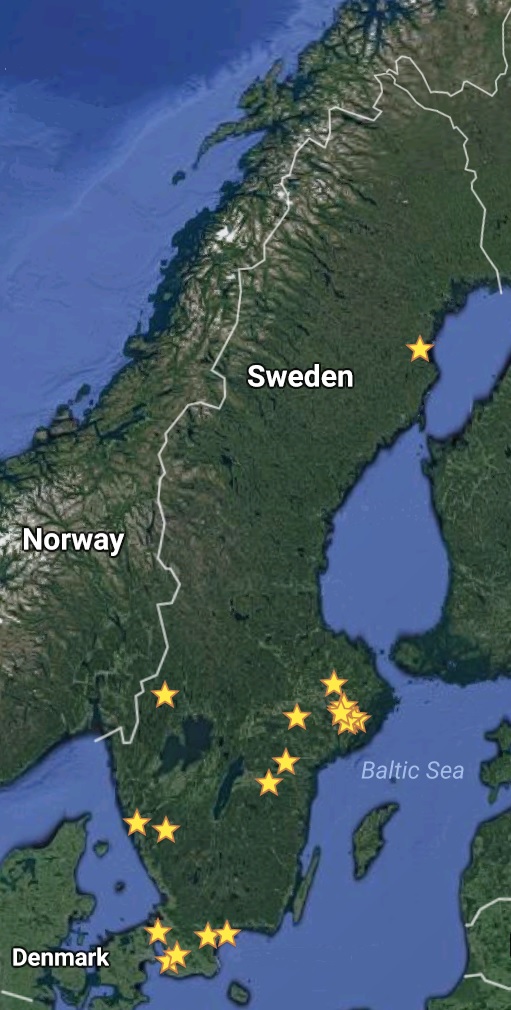
**
